# Clinical Observation of Extensively Hydrolysis Protein Formula With Feeding Intolerance in Preterm Infants

**DOI:** 10.3389/fped.2022.871024

**Published:** 2022-06-13

**Authors:** Liping Yin, Jingjing Ma, Heng Liu, Qianying Gu, Li Huang, Qi Mu, Ning An, LiJuan Qian, Lixing Qiao

**Affiliations:** ^1^Department of Pediatrics, Zhongda Hospital Affiliated to Southeast University, Nanjing, China; ^2^Department of Nuclear Medicine, Zhongda Hospital Affiliated to Southeast University, Nanjing, China; ^3^College of Pediatrics, Xinjiang Medical University, Ürümqi, China

**Keywords:** extensively hydrolysed formula, premature infants, feeding intolerance, whole intestinal nutrition, metabolic bone disease (MBD) of prematurity

## Abstract

**Objective:**

To investigate whether feeding extensively hydrolysis protein formula during the NICU hospitalization was more beneficial for preterm infants with a gestational age (GA) ≤34 weeks when breastfeeding was not possible.

**Methods:**

In total, 587 preterm infants were randomly divided into two groups: observation groups fed with extensively hydrolyzed formula (EHF) milk and control groups fed with standard preterm formula (SPF) milk until discharge from the neonatal intensive care unit (NICU). The incidence of complications during hospitalization was recorded in both groups. Then, two groups were uniformly fed with 0-to-6-month infant formula milk and followed-up for 6 months after discharge.

**Results:**

The final study included 370 premature infants, including 185 babies in the observation group and 185 in the control group. In contrast to the SPF, feeding EHF among preterm infants of GA <34 weeks during NICU hospitalization significantly reduced the incidence of feeding intolerance (FI) (14.1 vs. 30.3%, *p* < 0.01). The incidence of necrotizing enterocolitis (NEC) was significantly reduced in the observation group (2.2 vs. 6.5%, *p* < 0.05), but there was no significant difference in the incidence of other related complications. At discharge, there was no difference in total serum protein (46.6 vs. 46.4 g/L), albumin (33.5 vs. 34.2 g/L), and calcium (2.37 vs. 2.35 mmol/L), but the serum phosphorus concentrations associated with skeletal mineralization (2.10 vs. 2.22 mmol/L, *p* < 0.05) was significantly reduced and alkaline phosphatase significantly rose (254 vs. 220 IU/L, *p* < 0.05) in the observation group. No significant difference was found in the growth rates of body weight, head circumference, or body length, either during the NICU hospitalization or during the 6-month follow-up after discharge (*p* > 0.05).

**Conclusions:**

Feeding premature infants of GA ≤34 weeks with EHF reduced the incidence of FI, but had no advantage in establishing whole intestinal nutrition, shortening parenteral nutrition (PN) time, or hospitalization time. It had little effect on physical growth or development during NICU hospitalization and within 6 months after discharge. However, it may increase the incidence of metabolic bone disease (MBD).

## Introduction

According to the World Health Organization (WHO), the average incidence of premature birth is 11.1%. Approximately 14.9 million premature babies are recorded every year, whose mortality accounts for up to half of that of newborns (1). China has the largest population in the world, where 14–15% of children under 5 years of age died from preterm birth-related complications from 2000 to 2008 (2). Due to the immature digestive system of premature infants, feeding problems, such as feeding intolerance (FI), often lead to the interruption of enteral feeding, which is the main factor resulting in the extrauterine growth retardation (EUGR) in premature infants (3). Some studies have shown that premature birth survivors are at a higher risk of EUGR compared with their term counterparts in the near future (4, 5). Parenteral nutrition (PN) is needed when enteral nutrition (EN) cannot meet daily growth and development, while long-term PN will bring a series of problems, such as higher morbidity of sepsis and PN-associated cholesterol (PNAC), eventually resulting in an increased length of hospital stay.

There have been many consensuses on the management of early EN in preterm infants (6), such as breastfeeding, the successful establishment of total enteral feeding as soon as possible, and shortening the time of PN. Expressed breast milk is the best choice for feeding premature infants (7). When breast milk is insufficient or mothers are unable to breastfeed, the donor milk can be used. However, breast milk banks have not been established in the best part of hospitals in China so that most hospitalized infants are fed formula. The formula is blessed with more energy, protein, vitamins, and minerals than breast milk so formula-fed infants may grow faster than breastfeeding infants (8, 9). Nevertheless, excessive weight gain in the early neonatal period will add to the risk of metabolic diseases, such as obesity, diabetes, and cardiovascular disease in adulthood (10, 11). In addition, the incidence of FI, necrotizing enterocolitis (NEC), and total mortality are also higher in preterm infants fed by formula instead of mothers' or donors' milk (8, 12). Besides, interruption of enteral feeding is the predominant cause of EUGR in preterm infants (13). As a result, it is very important to choose powdered milk that is more suitable for the intestinal function of preterm infants with GA ≤34 weeks.

The extensively hydrolyzed formula is a special kind of milk powder in which proteins are hydrolyzed into short peptides and some amino acids through special processes (14). It is mainly recommended currently for babies with cow's-milk protein allergy (CMPA) and gastroesophageal reflux disease (GERD). More and more researchers have applied extensively hydrolyzed formula (EHF) for short bowel syndrome (15), FI, and NEC of preterm infants (16, 17). Regrettably, there has been no more evidence to prove that enteral feeding of EHF after birth can reduce the incidence of FI in premature infants, achieve total EN faster, shorten the process of PN, or the length of hospitalization (18, 19).

In this study, we enrolled premature infants who cannot breastfeed with GA ≤34 weeks. The research was aimed at whether feeding EHF during NICU hospitalization in premature infants within 34 weeks would decrease the incidence of FI. Besides, we also explored whether feeding EHF could speed up the time of total EN for premature infants, shorten the time of PN as well as hospital stays of NICU, and be beneficial for premature infants to defecate.

## Research Methods

### Patients

A total of 4,405 newborns were admitted to the Neonatal Intensive Care Unit (NICU) of Zhongda Hospital Southeast University from November 2014 to November 2017, among which premature infants (GA ≤34weeks) were 983 (22.3%). According to the inclusion criteria in the previous trial scheme, a total of 497 infants were enrolled with their parents agreeing and signing the informed consent. They were randomly divided into the observation group (extensively hydrolyzed protein formula milk feeding group), including 248 neonates, and the control group (formula for premature infants feeding group), including 249.

### Study Design

The detailed research methods of this study refer to the experimental schemes published in *Trials* by our research group in 2015 (20). This study has been reviewed by the Ethics Committee of Clinical Research of the Zhongda Hospital Southeast University, and the approval document of the Ethics Committee is 2014ZDSYLL115.0.

### Data Analysis

The data capture and analysis were done using the Statistical Package for Social Sciences (SPSS) version 22.0. The *F*-test was used to assess the homogeneity of variance of a normal distribution, and the results were presented as as MD ± SD, whereas the Mann–Whitney rank sum test was used to assess the heterogeneity of variance of a non-normal, and the results were expressed as MD (P25, P75). The counting data were described by case number and percentage, and analyzed by a chi-square test. The value of *p* < 0.05 was the accepted level of statistical significance, and *p* < 0.01 indicated extremely significant difference.

### Ethical Clearance

The Ethics Committee of Clinical Research of the Zhongda Hospital Southeast University provided the ethical clearance to conduct this study-(Ethical Review Committee Protocol ID No: 2014ZDSYLL115.0). Consent was obtained from children's parents before the study was administered. Permission was obtained from the management of the two hospitals. The data were anonymize to conceal the identity of patient. The analysis was conducted in a way that would not link the final results to individual patients.

## Results

### Patient Characteristics

**Figure 1:**
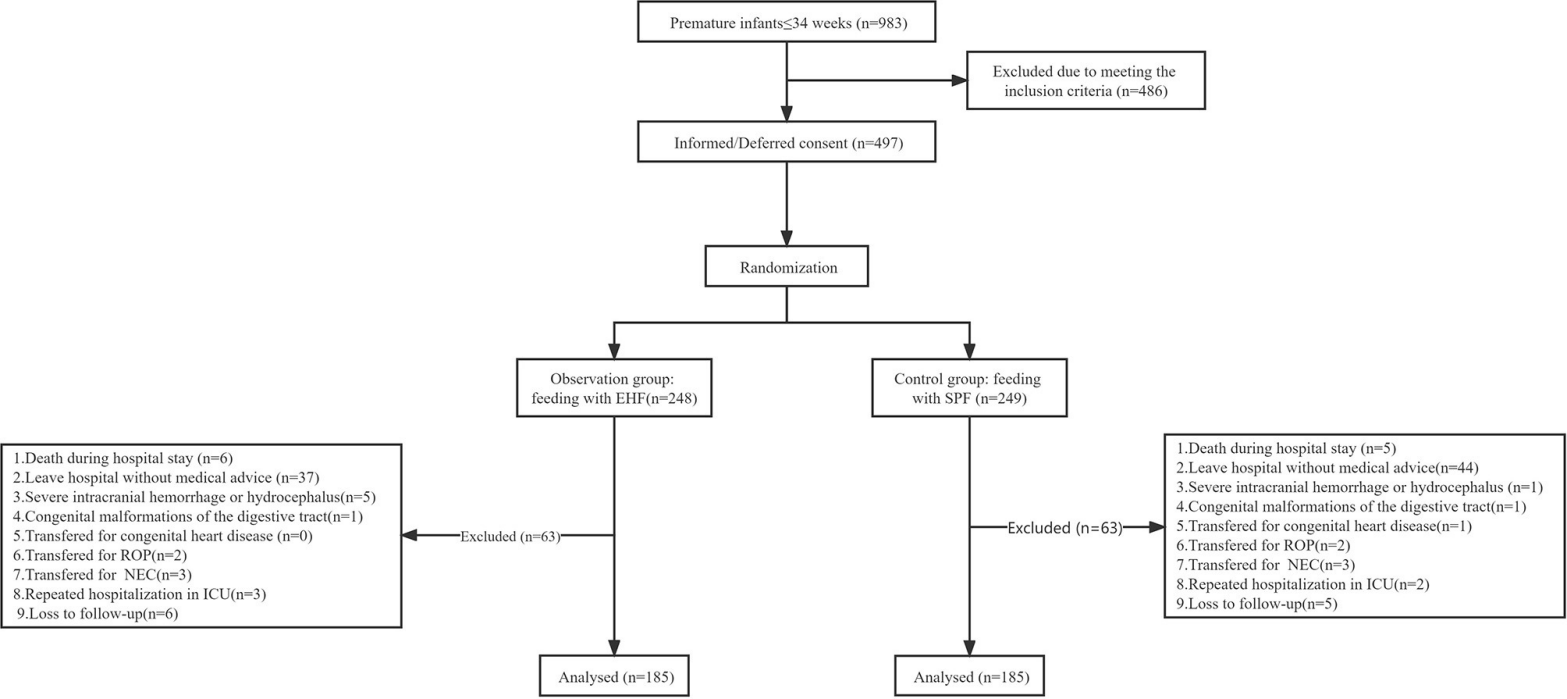
Study enrollment flow diagram.

In this study, 248 newborns were in the observation group (EHF) and 249 in the control group (standard preterm formula [SPF]). Strictly following exclusion criteria, 63 individuals from the observation group and 64 from the control group were excluded. Finally, 370 premature infants were studied, including 185 in the observation group and 185 in the control group as shown in Figure 1.

There were no significant differences in gender, gestational age, multiple pregnancy and delivery, *in vitro* fertilization (IVF), delivery method, 5-min Apgar score, admission age, small for gestational age (SGA) composition ratio (21), birth weight, birth length, and birth head circumference between the two groups (*p* > 0.05), but the first feeding time in the observation group was later than that in the control group (21 vs. 13 h, *p* < 0.05). Table 1 shows the characteristics of participants.

**Table 1:** Characteristics of premature infants between the two groups.

**Demographic characteristics**	**Observation group (*n* = 185)**	**Control group (*n* = 185)**	***X^**2**^/Z/t***	***P***
Gender (Male:Female)	104:81	99:86	*χ^2^ = 0.273*	*0.601*
Gestational Age (d)	228.0 (217.0, 234.0)	226.0 (219.0, 232.0)	*Z = −0.954*	*0.340*
Single:Multiple	136:49	138:47	*χ^2^ = 0.056*	*0.812*
Assisted reproduction (Yes:No)	28:157	17:168	*χ^2^ = 3.061*	*0.080*
Natural delivery:Cesarean section	97:88	104:81	*χ^2^ = 0.534*	*0.465*
Apgar score in 5 min (Yes:No)	9 (8, 10)	10 (9, 10)	*Z = −1.752*	*0.080*
Apgar score in 5 min ≤5 (Yes:No)	*5:180*	*5:180*	*χ^2^ = 0.000*	*1.000*
Admission age (h)	1.00 (1.00, 2.75)	1.00 (0.80, 2.00)	*Z = −1.268*	*0.205*
Birth weight (g)	1800.51 ± 398.31	1825.00 ± 404.81	*t = −0.586*	*0.558*
Weight at admission (g)	1791.35 ± 395.00	1808.92 ± 403.62	*t = −0.423*	*0.672*
Head circumference at admission (cm)	29.70 (28.00, 31.00)	30.00 (28.25, 30.95)	*Z = −0.210*	*0.833*
Body length at admission (cm)	42.50 (41.00, 44.15)	42.00 (41.00, 44.05)	*Z = −0.495*	*0.621*
SGA *n* (%) ^*^	13 (7.0%)	19 (10.3%)	*χ^2^ = 1.232*	*0.267*
First feeding time (h)	21.00 (10.00, 38.00)	13.00 (3.40, 23.50)	*Z = −5.091*	*0.000*

* *Small for gestational age (SGA) pointed out that the birth weight was lower than the 10th percentile of the same gestational age and same gender, and the judgment criteria was made from reference*.

### Primary Outcome

In this study, the total incidence of FI in 370 premature infants was 22.2%. The incidence of FI in the observation group (14.1%) was significantly lower than that in the control group (30.3%), as *p* < 0.01 shown in Table 2.

**Table 2:** Comparison of incidence of feeding intolerance (FI) between the two groups.

			**Feeding intolerance**		**Total**	***χ^2^***	***P***
			**Occurred**	**Non-occurred**			
Group	Observation	*n* (%)	26 (14.1%)	159 (85.9)	185		
	Control	*n* (%)	56 (30.3%)	129 (69.7%)	185		
Total		*n* (%)	82 (22.2%)	288 (77.8%	370	14.101	0.000

Logistic regression model analysis of FI showed that extensively hydrolyzed protein formula could effectively avoid FI in premature infants (odds ratio [OR] 0.297 [95% *CI* 0.167–0.527]). Whole milk protein formula for premature infants, small gestational age, multiple pregnancy and delivery, IUGR, and late first milking time after birth are all high-risk factors for FI, as shown in Tables 3, 4.

**Table 3:** Indicators of possible influencing factors for FI.

**Contents**		**Group with FI**	**Group without FI**	***χ^2^/Z***	***P***
Group	deep-hydrolyzed protein formula	26	159	14.101	0.000
	formula for premature infants	56	129		
Gender	Male	42	161	0.565	0.452
	Female	40	127		
Multiple pregnancy	Single	53	221	4.865	0.027
	Multiple	29	67		
Delivery	Natural delivery	50	151	1.878	0.171
	Cesarean section	32	137		
Apgar score in 5 min	≤5	3	7	0.366	0.545
	> 5	79	281		
Assisted reproduction	Yes	12	33	0.603	0.438
	No	70	255		
SGA at birth	Yes	16	16	15.736	0.000
	No	66	272		
Gestational age (d)		221 [210, 226]	229 [220, 234]	−5.570	0.000
Admission age (h)		1.0 [1.0, 2.0]	1.0 [0.8, 2.0]	−0.415	0.678
First milking time (h)		19.00 [9.75, 42.00]	17.25 [5.00, 28.00]	−2.219	0.026
Birth weight (g)		1480.0 [1350.0, 1792.5]	1880.0 [1600.0, 2137.5]	−6.505	0.000

**Table 4:** Logistic regression analysis of FI risk factors.

**Independent variables**	**Given value**	**β**	**Walds χ^2^**	***P***	**OR**	**OR95%CI**
Group	deep-hydrolyzed protein formula = 1 formula for premature infants = 2	−1.214	17.211	0.000	0.297	0.167–0.527
Gestational age		0.073	27.453	0.000	1.076	1.047–1.106
Multiple pregnancy	Single = 1 Multiple = 2	−0.715	5.414	0.020	0.489	0.268–0.893
First milking time		−0.017	4.147	0.042	0.984	0.968–0.999
SGA	Yes = 1 No = 2	1.888	19.141	0.000	6.606	2.835–15.391

### Secondary Outcome

Nutrients in the EHF in the observation group were not higher than those in the control group. This study found that the duration of parenteral intravenous nutrition, total EN, and hospital stay in the observation group were all longer than those in the control group, but *p* > 0.05 with no statistical significance. The average daily defecation frequency of preterm infants in the observation group was higher than that in the control group, but *p* was also more than 0.05 with no statistical significance. These data are shown in Table 5.

**Table 5:** Comparison of duration of enteral nutrition (EN), hospital stay, and defecation between the two groups of premature infants.

**Group (*n*)**	**Total enteral**	**Intravenous nutrition**	**Average number of**	**The average number**	**Hospitalized**
	**nutrition time (d)**	**time (d)**	**bowel movements per day**	**of enemas required per day**	**duration (d)**
Observation (185)	10 (7, 14)	10 (7, 14)	2.1 (1.6, 2.8)	0.20 (0.10, 0.40)	23.00 (15.50, 33.50)
Control (185)	9 (7, 14)	9 (6,17)	2.0 (1.5, 2.9)	0.20 (0.10, 0.30)	21.00 (15.00, 31.00)
*Z*	–0.013	–0.132	–0.937	–1.398	–1.338
*P*	0.989	0.895	0.349	0.162	0.181

The incidence of NEC in the observation group was significantly lower than that in the control group (*p* < 0.05), which was statistically significant. Although the PN duration in the observation group was slightly longer than that in the control group, the incidence of cholestasis and PNAC did not increase, and the values of *p* were both above 0.05. The incidences of EUGR were significantly increased in the other two groups, but there was no statistical significance between the two groups (*p* > 0.05). These are shown in Table 6.

**Table 6:** Comparison of the incidence and composition of parenteral nutrition(PN)-related diseases in the two groups of premature infants during neonatal intensive care unit (NICU) hospitalization.

**Group (*n*)**	NEC^*^	**Cholestasis**	PNAC^*^	**Thyroid dysfunction**	EUGR^*^when discharge	**New EUGR when discharge**
	***n* (%)**	***n* (%)**	***n* (%)**	***n* (%)**	***n* (%)**	***n***
Observation (185)	4 (2.2%)	15 (8.1%)	10 (5.4%)	60 (32.4%n)	67 (36.2%)	54
Control (185)	12(6.5%)	8 (4.3%)	8 (4.3%)	56 (30.3%)	66 (35.7%)	47
*χ^2^*	4.181	2.272	0.234	0.201	0.012	0.866
*P*	0.041	0.132	0.629	0.654	0.914	0.352

* *NEC, Necrotising enterocolitis; PNAC, parenteral nutrition associated cholestasis; EUGR, extrauterine growth retardation; the judgment criteria was made from reference*.

Before discharge, there was no difference of serum levels of total protein, albumin, and total calcium between the two groups, but serum phosphorus in the observation group was significantly lower than that in the control group, while alkaline phosphatase was significantly higher than that in the control group (*p* < 0.05). These are shown in Table 7.

**Table 7:** Comparison of serological examinations before discharge between the two groups of premature infants.

**Group (*n*)**	**Total protein (g/L)**	**Albumin (g/L)**	**Calcium (mmol/L)**	**Phosphorus (mmol/L)**	**ALP (IU/L)**
Observation (185)	46.60 (43.40, 50.35)	33.50 (31.80, 36.00)	2.37 (2.26, 2.46)	2.10 (1.84, 2.34)	254.00 (203.50, 334.50)
Control (185)	46.40 (43.95, 50.00)	34.20 (32.00, 36.00)	2.35 (2.22, 2.45)	2.22 (1.92, 2.58)	220.00 (178.00, 278.00)
*Z*	−0.479	−1.285	−1.758	−1.758	−3.748
*P*	0.632	0.199	0.079	0.006	0.000

At discharge, there were no differences of the body weight, body length, and head circumference between patients of the two groups. The growth rate of body weight, body length, and head circumference during hospitalization between the two groups showed no distinction with *p* > 0.05, as shown in Table 8.

**Table 8:** Comparison of growth and development indexes at discharge and growth rate during hospitalization.

**Group (n)**	**Weight when**	**Body length when**	**Head circumference**	**Rate of weight**	**Growth rate of**	**Growth rate**
	**discharge (kg)**	**discharge (cm)**	**when discharge (cm)**	gain^*^g/(kg.d)	**body length**	**of head**
	**MD (25–75%)**	**MD (25–75%)**	**MD (25–75%)**		**(cm/w)**	**circumference (cm/w)**
Observation (*n* = 185)	2,140 (1,990, 2,375)	46.00 (44.75, 47.20)	31.90 (31.00, 32.50)	7.49 (4.44, 9.76)	0.91 (0.81, 1.11)	0.58 (0.50, 0.70)
Control (*n* = 185)	2,110 (1,950, 2,325)	45.50 (44.00, 47.00)	31.50 (30.75, 32.50)	7.38 (4.29, 9.70)	0.89 (0.70, 1.13)	0.55 (0.45, 0.70)
*Z/t*	−1.531	−1.759	−1.287	−0.508	−0.994	−1.605
*P*	0.126	0.079	0.198	0.611	0.320	0.108

* *Calculation formula of weight growth rate*.

**Figure 2:**
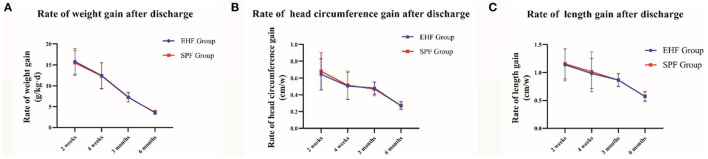
**(A–C)** Growth and development follow-up after discharge.

Growth and development follow-up were conducted, respectively at 2 weeks, 4 weeks, 3 months, and 6 months after discharge. It was found that there was no significant difference of weight, body length, and head circumference growth rate of premature infants between the two groups, with all values of *p* < 0.05, as shown in Table 9 and Figure 2.

**Table 9:** Comparison of the growth and development indexes of the two groups of premature infants after discharge from hospital.

**Follow-up time**	**Indicators g/(kg.d) or cm/w**	**Group**		***t/Z***	***P***
		**Observation (*n* = 185)**	**Control (*n* = 185)**		
2 weeks	Weight MD (25–75%)	15.80 (14.01,17.48)	15.53 (14.24,16.79)	−0.934	0.350
	Body length MD (25–75%)	1.15 (1.00,1.30)	1.15 (1.00,1.30)	−0.412	0.681
	Head circumference MD (25–75%)	0.65 (0.55,0.75)	0.65 (0.55,0.80)	−1.895	0.058
4 weeks	Weight MD ± SD	12.46 ± 3.05	12.39 ± 3.13	0.233	0.816
	Body length MD (25–75%)	0.95 (0.80,1.15)	1.00 (0.83,1.15)	−0. 686	0.493
	Head circumference MD (25–75%)	0.50 (0.40,0.60)	0.50 (0.40,0.60)	−0.664	0.507
3 months	Weight MD (25–75%)	7.30 (6.59,7.84)	7.15 (6.52,7.95)	−0.670	0.503
	Body length MD (25–75%)	0.87 (0.80,0.93)	0.86 (0.79,0.93)	−0.587	0.557
	Head circumference MD (25–75%)	0.47 (0.44,0.52)	0.47 (0.42,0.51)	−1.334	0.182
6 months	Weight MD (25–75%)	3.50 (3.30,3.80)	3.60 (3.30,4.00)	−1.695	0.090
	Body length MD ± SD	0.57 ± 0.08	0.57 ± 0.09	−0.146	0.884
	Head circumference MD (25–75%)	0.26 (0.24,0.30)	0.26 (0.25,0.30)	−0.745	0.456

## Discussion

The present study is a prospective, randomized, single-blinded, single-center trial,totally divided into two stages. The first stage is to study the FI incidence rate of premature infants fed with two formulas of milk during NICU hospitalization. Then, we explored for parenteral vs. EN and spontaneous defecation functions of premature infants. In the second stage, all subjects were followed-up for 6 months to observe their nutrition, growth, and development.

Compared with SPF, feeding EHF during NICU stay in preterm infants within 34 weeks can significantly reduce the incidence of FI. Premature infants born small for GA (<34 weeks) are prone to FI due to immature gastrointestinal development. First, the intestinal villi are dysplastic, the intestinal absorption area is reduced, and the intestinal nutrition absorption is insufficient (22). Second, premature infants' gastrointestinal peristalsis ability is also very weak, causing prolonged gastric emptying time (23).

Di Mauro A et al. found that nearly 75% of very low birth weight (VLBW) will suffer from FI (24). Similarly, our study found that the incidence rate of FI in premature infants (<34 weeks), 14.1% in the EHF group and 30.3% in the SPF group. Nowadays, many studies have found high-risk factors associated with FI as follows. (1) Low gestational age, low birth weight, asphyxia, respiratory distress, and enteral feeding delay (6); (2) premature infant formula feeding (8, 12); (3) gut microbiota affected by early antibiotic using (25); (4) applied Indomethacin or Ibuprofen in the treatment of hemodynamically significant patent ductus arteriosus (hsPDA) (26); and (5) cow's-milk protein intolerance (CMPI): a subset of premature infant formula fed will develop to FI or recurrence NEC-like illness, such as vomiting, diarrhea, and hematochezia (27, 28). It leads to repeated intestinal feeding interruptions and then requires multiple courses of PN. Symptoms improve soon after administration of deeply hydrolyzed protein formula or amino acid dried milk. Preterm infants as described above need to consider FI caused by CMPI.

The present study found that, in addition to the above-mentioned high-risk factors, preterm infants delivered in multiple pregnancies were also found to be high-risk factors for FI, which may be associated with the fetus in multiple pregnancies being prone to preterm delivery earlier. Meanwhile, the present study found that preterm infants with SGA were more likely to develop into FI. Possibly related to the following factors: the infant's postnatal gastrointestinal tract (GIT) is also involved in the developmental maturation of the immune system, and the premature infant's GIT is developmentally immature for intrauterine growth retrieval (IUGR), leading to diminished protection against food allergens and environmental micro-organisms (29). GIT plays an important role in the development of neonatal immune system. Premature infants with intrauterine growth retardation have an immature GIT, which leads to diminished protection against food allergens and environmental micro-organisms (30). At the same time, the small intestine weight, length, intestinal wall thickness, length and number of the intestinal villous, and crypt depth are reduced, causing impaired nutrient absorption and utilization. In conclusion, neonates with intrauterine growth retardation are not only at a higher incidence of postnatal FI but also at a higher risk of developing NEC (31).

Interestingly, due to the inability to conduct follow-up, IIA proven NEC (mildly ill) and more serious cases are excluded from the study, and the incidence of total NEC did not differ between the two groups. A systematic review completed by Derek Hang Cheong Ng and others also corroborated this result (18). However, in this study, the incidence of NEC decreased significantly (2.2 vs. 6.5%, *p* < 0.05), which may be related to the inclusion of only stage 1 NEC in our study. A study reveals that semi-elemental or elemental formulas may be an effective nutritional intervention to reduce the risk of NEC in preterm infants. The nutrients in semi-elemental or elemental formulas are easy to absorb, which is expected to reduce stress on the gut and potentially avoid the proinflammatory processes that lead to NEC (32). For severe NEC, complicated infections may be associated, so deep hydrolyzed milk is less effective in prevention. This interesting result may be a direction worthy of further investigation.

The current abundance of prevention and treatment measures for FI. (1) Breastfeeding is the best option and preterm infants who cannot be fed with EHF (12, 16). In our research, EHF-fed preterm infants had a significantly lower FI incidence than in the SPF group (14.1 vs. 30.3%, *p* < 0.05), but some studies suggest that there is no basis to show that enteral EHF initiation after the birth of preterm infants reduces the incidence of FI (18). (2) EN was implemented as soon as possible. Our study found that the incidence of FI in premature infants with early EN (average time = 19.00 h) was lower than that of those who had started EN late (average time = 17.25 h) (*OR*:0.984,95% *CI*:0.968–0.999).

Our study found that, compared with SPF feeding, enteral feeding of EHF immediately after birth in preterm infants (≤34 weeks) did not decrease the time to full EN. There was no significant difference between the two groups in PN time, hospital stay, time to restore birth weight, and defecation during hospitalization. This may be related to the significantly lower nutrient components of EHF, energy provided per 100 ml and protein/energy ratio than SPF. It was related to the fact that the nutrient content, energy provided per 100 ml, and protein/energy ratio in EHF were all significantly lower than in SPF. Although some studies have found that increasing protein intake during parenteral or EN cannot improve the physical growth and neural development of preterm infants (birth weight 500–1,249 g) during NICU hospitalization and 0–2 years old (33). However, the latest study found that SPF-fed preterm infants could reach full enteral feeding in a shorter period of time (10 vs. 14 days) and reduce the duration of PN and hospital stay compared with EHF (18, 19). Therefore, the nutritional safety of enteral feeding of EHF started immediately after birth in preterm infants (≤34 weeks) needs to be confirmed by more studies and longer follow-up.

The guidelines from the European Society for Gastrointestinal Nutrition and Hepatology (ESPGHAN) recommended (21). (1) The daily energy demand of preterm infants is 110–135 kcal/(kg·d). (2) The protein-to-energy (P/E) ratio is the (BW <1,000 g, P/E 3.6–4.1 g/100 kcal, BW between 1,000 and 1,800 g, P/E 3.2–3.6 g/100 kcal). (3) The calcium requirement is 120–140 mg/(kg·d) or 110–130 mg/100 kcal, and the phosphorus requirement is 60–90 mg/(kg·d) or 55–80/100 kcal. Whereas, the EHF in our study provided only 66 kcal/100 ml, the P/E ratio was only 2.42 g/100 kcal, and the calcium phosphorus content (calcium content 71.2 mg/100 kcal, phosphorus content 39.4 mg/100 kcal) was significantly lower than that of the SPF (calcium content 125 mg/100 kcal, phosphorus content 70 mg/100 kcal), which all have the potential to affect the nutrition and growth and development of preterm infants in the near or long term, and we have shown in further studies, and certain measures have been taken to avoid these deficiencies, such as changing the EHF to a more nutrient rich SPF after reaching total EN, or timely addition of various nutrients when the EHF is fed.

In conclusion, feeding EHF to preterm infants (GA <34 weeks) during NICU hospitalization reduces the incidence of FI. Low gestational age, delayed enteral feeding, formula feeding for preterm infants, multiple gestational delivery, and SGA at birth are high risk factors for FI in preterm infants. However, compared with SPF, EHF did not have advantages in establishing total EN, reducing the duration of PN, reducing the length of hospital stay, restoring birth weight more quickly, and improving bowel movements during hospitalization.

## Data Availability Statement

The original contributions presented in the study are included in the article/supplementary material, further inquiries can be directed to the corresponding author/s.

## Ethics Statement

The studies involving human participants were reviewed and approved by the Ethics Committee of Clinical Research of the Zhongda Hospital Southeast University. Written informed consent to participate in this study was provided by the participants' legal guardian/next of kin.

## Author Contributions

LQia and LY: conceptualization. LY: validation. LH and LQian: methodology. QM and NA: investigation and formal analysis data. JM and QG: curation. HL: writing—original draft preparation. LQia: supervision and project administration. All authors have read and agreed to the published version of the manuscript.

## Conflict of Interest

The authors declare that the research was conducted in the absence of any commercial or financial relationships that could be construed as a potential conflict of interest.

## Publisher's Note

All claims expressed in this article are solely those of the authors and do not necessarily represent those of their affiliated organizations, or those of the publisher, the editors and the reviewers. Any product that may be evaluated in this article, or claim that may be made by its manufacturer, is not guaranteed or endorsed by the publisher.
